# The Role of the p16 and p53 Tumor Suppressor Proteins and Viral HPV16 E6 and E7 Oncoproteins in the Assessment of Survival in Patients with Head and Neck Cancers Associated with Human Papillomavirus Infections

**DOI:** 10.3390/cancers15102722

**Published:** 2023-05-11

**Authors:** Andrejs Lifsics, Maksims Cistjakovs, Liba Sokolovska, Renars Deksnis, Modra Murovska, Valerija Groma

**Affiliations:** 1 Department of Otorhinolaryngology, Riga Stradiņš University, Pilsoņu 13, LV-1002 Riga, Latvia; 2 Institute of Microbiology and Virology, Riga Stradiņš University, Rātsupītes 5, LV-1067 Riga, Latvia; 3 Institute of Anatomy and Anthropology, Riga Stradiņš University, Kronvalda blvd 9, LV-1010 Riga, Latvia

**Keywords:** oropharynx, larynx, hypopharynx, squamous cell carcinoma, HPV, PCR, immunohistochemistry, p16, p53, E6/E7 viral oncoproteins, survival analysis

## Abstract

**Simple Summary:**

The role of human papillomavirus (HPV) in the survival of patients with head and neck squamous cell carcinoma (HNSCC) is an important topic. The recognition of additional markers could play a significant role in survival prognosis. Our study aimed to assess the roles of different molecular and immunohistochemical factors in the survival of patients with HNSCC. We analyzed 106 HNSCC samples and confirmed the roles of HPV DNA and p16, p53, and HPV16 E6 and E7 proteins in different subgroups of HNSCC. In addition to p16, the immunohistochemical overexpression of HPV16 E6 protein should be used for patient survival prognosis.

**Abstract:**

The role of HPV in the survival prognosis of patients with head and neck squamous cell carcinoma, especially patients with laryngeal squamous cell carcinoma (LSCC) and hypopharyngeal squamous cell carcinoma (HPSCC), is still somewhat ambiguous. The present study aimed to explore the significance of tumor suppressor proteins and HPV16 E6 and E7 oncoproteins in the assessment of survival in patients with oropharyngeal squamous cell carcinoma (OPSCC), LSCC, and HPSCC associated with high-risk (HR-) and low-risk (LR-) HPV infections. By utilizing molecular and immunohistochemical investigations of HNSCC samples and patient data, univariate and multivariate survival analyses were conducted. The presence of HPV DNA (LR- and HR-HPV) was associated with a better 5-year OS and DSS for OPSCC and LSCC. The IHC overexpression of HPV16 E6 protein and p16 protein was associated with better survival in the univariate (for OPSCC) and multivariate (OPSCC and HPSCC) survival analyses. The overexpression of p53 was associated with better survival in OPSCC. HPV infection plays a significant role in the tumorigenesis of HNSCC, and the immunohistochemical assessment of HPV16 E6 protein expression should be interpreted as a useful prognostic marker for OPSCC and HPSCC.

## 1. Introduction

As one of the most common cancers globally, HNSCC accounts for more than 660,000 new cases and 325,000 deaths annually [1]. According to the GLOBOCAN data, 98,412 new cases of OPSCC, 98,412 new cases of LSCC, and 84,254 new HPSCC were registered in 2020 [2].

LR-HPV types encompass the majority of known HPV (more than 200) types and are not usually associated with cancer development [3]. By contrast, HPV types 16, 18, 31, 33, 35, 39, 45, 51, 52, 56, 58, 59, 66, and 68 are viewed as HR-HPV types. HR-HPV infection has long been recognized as an etiologic factor of anogenital cancers and has also relatively recently been recognized as an etiologic factor of some head and neck cancers. While HR-HPV infection and HPV-16 are most commonly strongly linked to OPSCC development (with HPV prevalence ranging from 45 to 90%) [4], in other head and neck cancers such as LSCC and HPSCC, the role of HR-HPV is still debated, as these cancers tend to be HPV-negative more frequently and are studied less frequently when compared to OPSCC.

HPV-positive head and neck cancers seem to be distinct from their HPV-negative counterparts in various aspects from the molecular mechanisms of transformation and tumor progression to epidemiology and, importantly, patient survival. The HPV status in squamous cell carcinomas overall has been demonstrated to be a prognostic factor for survival, and HPV-associated OPSCCs specifically are associated with a reduced risk of death and a reduced risk of recurrence [5], yet for other head and neck squamous cell carcinomas (HNSCC) (e.g., LNSCC and HPSCC), such an association has not been confidently established [6,7,8,9,10,11,12,13,14]. Additionally, HPV-positive head and neck cancers have several molecular signatures: degradation of wild-type p53 and a lack of mutations in the p53-encoding gene, decreased expression of pRb, and subsequent increased expression of p16. These molecular differences could help distinguish HPV-associated cancers, thus aiding in treatment adjustment, and could serve as prognostic markers [5].

The oncogenic potential of HPV is mainly dependent on two of its early proteins: E6 and E7. These viral proteins interact with important cell cycle regulators (tumor suppressors) of the infected epithelial cells, causing their uncontrolled proliferation. Since HPV oncoprotein expression is considered necessary for carcinogenesis as well as causality, their expression could serve as a prognostic marker. Some researchers suggest that HPV-related head and neck cancers have a better prognosis due to a more aggressive and specific immune response to tumor-expressing HPV antigens, including E6 and E7. Some studies have demonstrated that T cells from patients with OPSCC proliferate and synthesize inflammatory cytokines upon HPV16 E6 and E7 oncoprotein recognition, and T cells from patients with HPV-related head and neck cancer show increased responses to E7 epitopes [6,15,16,17].

One of the oncoproteins, E6, promotes proteasomal p53 degradation via E6-associated ubiquitin ligase, thus deregulating cell cycle checkpoints, avoiding apoptosis, and inactivating one of the p53 targets, p21, which prevents cells from entering the S phase via cell cycle arrest in the G1 phase [18]. In non-HPV-associated cases of head and neck cancers, the p53-encoding gene is often mutated, resulting in a loss of p53 function or even gain of functions that promote invasion, metastasis, and cancer cell proliferation [19]. Studies have shown that patients whose HNSCCs are positive for HPV and lack p53 expression (due to p53 degradation via E6) have a better prognosis and better overall survival [20].

E7, on the other hand, strongly binds pRb and induces its proteasomal degradation, thus releasing a transcription factor called E2F, which again drives the cells to enter the S phase of the cell cycle [21,22]. Another consequence of the E7-mediated pRb degradation is the overexpression of p16, a potent tumor suppressor. The detection of p16 overexpression has been adopted as a molecular hallmark of HPV-associated OPSCCs, with studies demonstrating its positive effect on patient survival. Studies have demonstrated its positive effects on patient survival in OPSCC. For other HNSCCs of non-oropharyngeal subsites, such an association has not been established [23,24], with studies reporting a lack of p16 expression, even in the presence of HPV mRNA, or similar p16 expression levels, regardless of HPV positivity [25,26].

This study aimed to explore the significance of various molecular and cellular markers of HR-HPV and LR-HPV in the assessment of survival in patients with OPSCC, LSCC, and HPSCC.

## 2. Materials and Methods

### 2.1. Patients’ Characteristics

A total of 106 patients (95 (89.6%) males and 11 (10.4%) females) with histologically confirmed OPSCC, LSCC, and HPSCC treated at the Latvian Oncology Centre between January 2015 and August 2019 were enrolled in the study.

The sex, age, TNM stage, differentiation grade (G) of the tumor, smoking and drinking habits at the time of presentation, and treatment modalities were assessed for each patient. The survival data were gathered from The Centre for Disease Prevention and Control on 1 January 2022. In total, 34 of 106 patients had OPSCC, 41 had LSCC, and 31 had HPSCC (Table 1).

### 2.2. DNA Extraction

Fresh frozen cancer tissues (24 OPSCC, 34 LSCC, and 2 HPSCC) or formalin-fixed, paraffin-embedded (FFPE) tumor tissue blocks (10 OPSCC, 28 HPSCC, and 8 LSCC) were used to extract DNA material for further investigation.

The DNA extraction from fresh frozen tissue material was performed with the standard phenol/chloroform extraction method.

FFPE cancer samples were processed using a blackPREP FFPE DNA Kit (Analytik Jena, Germany) following the manufacturer’s protocol. To avoid cross-contamination, separate sterile blades were used for each specimen.

To assess the concentration and quality of the extracted DNA, a spectrophotometric analysis was performed (Nanodrop ND-1000 Spectrophotometer, Thermo Fisher Scientific, Waltham, MA, USA). Beta-(β-) globin was used as a quality control for the isolated DNA [27]. Only β-globin-positive samples were included in the further investigation of the gathered specimens.

### 2.3. RNA Extraction

Fresh frozen cancer tissue materials (24 OPSCC, 34 LSCC, and 2 HPSCC) or FFPE cancer tissue blocks (10 OPSCC, 28 HPSCC, and 8 LSCC) were processed for total RNA extraction.

Standard RNA extraction with TRIzol LS Reagent from Thermo Fisher Scientific was accomplished for fresh frozen tissue specimens according to the producer’s manual.

A PureLink FFPE Total RNA Isolation Kit (Thermo Fisher Scientific, USA) was used for RNA extraction from FFPE cancer samples, following the manufacturer’s protocol. Each sample was sectioned separately with a new sterile blade.

A spectrophotometric analysis was used to assess the concentration and quality of the extracted RNA.

### 2.4. HPV DNA Detection Using MY09/11 and GP5+/6+ Consensus Primers

A polymerase chain reaction (PCR) with the consensus primers MY9/MY11 and GP5+/6+ was used for the initial detection of the broad range of HPV types (HR-HPV and LR-HPV types) [28,29]. Electrophoresis in a 1.7% ethidium bromide gel was used to assess the PCR results. Amplification products of appropriate lengths for the primers that were used were considered HPV-positive. Each reaction included positive and negative controls.

### 2.5. HPV Genotyping

Consensus PCR-positive samples were further subjected to HPV genotyping. Primers for HPV 16 and 18 (L1) and the Anyplex II HPV28 multiplex real-time-PCR (RT-PCR) were used for HPV genotyping.

The results were visualized via electrophoresis in 1.7% agarose gel with an assessment of appropriate amplification products [29]. Positive and negative controls were used in each reaction.

Anyplex II HPV28 multiplex RT-PCR was used following the manufacturer’s recommendations (Seegene, Seoul, Republic of Korea).

### 2.6. HPV16 E6/E7 mRNA Detection

The detection of E6/E7 mRNA was performed using real-time PCR with the PreTect HPV-Proofer kit. The PreTect HPV-Proofer assay qualitatively detected the presence of HPV E6/E7 oncogene mRNA from HPV types 16, 18, 31, 33, and 45. It had an intrinsic sample control to assess specimen quality. Specimens with positive intrinsic controls were considered valid. Only HR-HPV-positive samples were used for E6/E7 mRNA detection.

### 2.7. Immunohistochemistry

OPSCC, LSCC, and HPSCC specimens were further processed as FFPE samples. The expression of HPV16 E6/E7 proteins, p53, and p16 proteins were assessed immunohistochemically.

For this purpose, 4–5 µm-thick FFPE tumor sections were mounted on SuperFrost Plus slides (Gerhard Menzel GmbH, Braunschweig, Germany) (Gerhard Menzel GmbH, Braunschweig, Germany). We used a previously tested and approved IHC protocol [30,31].

Briefly, after the standard preparation process, the sections were incubated overnight with the primary antibodies at 4 °C. We used a monoclonal mouse anti-CDKN2A/p16INK4a antibody (Abcam, Cambridge, UK, 1:300 dilution, ab201980); a monoclonal mouse anti-p53 antibody (Santa Cruz Biotechnology, Inc., Dallas, TX, USA, 1:50 dilution, sc-47698); a monoclonal mouse anti-HPV16 E6 + HPV18 E6 antibody (Abcam, Cambridge, UK, prediluted, ab51931) [32,33,34]; and a monoclonal mouse anti-HPV16 E7 antibody (Santa Cruz Biotechnology, Inc., 1:50 dilution, sc-6981). We used a HiDef Detection HRP Polymer system and a diaminobenzidine tetrahydrochloride substrate kit (Cell Marque, Rocklin, CA, USA) to visualize the products of IHC reactions. Counterstaining of cell nuclei within a tumor section with Mayer’s hematoxylin was used. In the negative controls of reactions, primary antibodies were omitted. The reaction results were assessed by two independent experienced investigators without knowledge of the clinical and molecular virology data.

The immunopositive reaction resulted in the appearance of brown reaction products using the anti-CDKN2A/p16INK4a, anti-p53, anti-HPV16 E6 + HPV18 E6, and anti-HPV16 E7 antibodies. Specifically, solely nuclear, combined nuclear and cytoplasmic immunostaining was observed when detecting p53 and HPV16 E7 proteins, p16 protein and HPV16 E6 protein, respectively.

A cut-off at 50% positive tumor cells for the p16 immunostaining was used, as proposed by Hong et al. (2013) [35].

The assessment of immunostaining for p53 was performed semiquantitatively. We considered a sample to be p53-postive (p53+) when the criteria described by Halec et al. (2013) were met [36]. The p53 overexpression (upregulation) was considered when p53 positivity was confirmed in >50% of tumor cells with intensity = 2 or >25% of tumor cells with intensity = 3. All FFPE specimens that did not reach these criteria were considered p53-negative (p53-; downregulation).

As all HR-HPV-positive specimens contained HPV16 DNA, only those were used for the IHC detection of E6 and E7 proteins. The IHC reaction results for the E6 and E7 viral proteins were estimated semiquantitatively in 20 randomly selected visual fields of each sample including the tumor and the surface epithelium of the regions of interest. To achieve enough statistical power, we used the expression levels of E6 and E7 at <10% as negative and at ≥10% as positive.

### 2.8. Statistical Data Analysis

All statistical analyses were performed using GraphPad Prism 9 (GraphPad Software, La Jolla, CA, USA). A standard statistical analysis was performed to assess the data distribution. A nonparametric Spearman’s correlation analysis was used to find any correlations between the groups [37]. A univariate survival analysis was performed using the Kaplan–Meier method; overall and disease-specific survivals (OS and DSS) were assessed. A multivariate survival analysis was performed using the Cox regression method. *p* values less than 0.05 (*p* < 0.05) were considered statistically significant.

## 3. Results

### 3.1. HPV DNA Analysis

We analyzed HNSCC samples for HPV DNA sequences and genotypes. HPV DNA was found in 92/106 (86.79%) of the HNSCC samples. More specifically, it was found in 29/34 (85.29%) of the OPSCC samples, 32/41 (78.05%) of the LSCC samples, and 31/31 (100%) of the HPSCC samples. The predominant genotype was HPV16, which was confirmed in 68/106 (65.09%) of the HNSCC samples. More precisely, it was found in 26/34 (76.47%) of the OPSCC samples, 22/41 (53.66%) of the LSCC samples, and 20/31 (64.52%) of the HPSCC samples. In 7/106 of the HNSCC samples, we detected HPV coinfections. In addition to HPV16 DNA, we found HPV31 (2 of 7), 33 (1 of 7), 35 (1 of 7), and 56 (4 of 7). Our further analysis was therefore focused on HPV16 due to its high prevalence.

### 3.2. HPV16 E6/E7 mRNA Expression

HPV16-postive HNSCC samples were analyzed for the presence of HPV16 E6/E7 mRNA (E6/E7 mRNA+). We detected HPV16 E6/E7 mRNA in 15/26 (57.7%) of the OPSCC samples, 2/22 (9%) of the LSCC samples, and 0/20 of the HPSCC samples. A correlation analysis of the semiquantitative HPV16 viral load results and the presence of HPV16 E6/E7 mRNA showed a moderate positive correlation (Sr = 0.601, *p* < 0.0001). Moreover, a weak positive correlation was found between p16 overexpression and E6/E7 mRNA expression (Sr = 0.472, *p* < 0.0001). Simultaneously, no correlation between p53 downregulation (p53−) and E6/E7 mRNA expression was found.

### 3.3. IHC Expression of p16 in HNSCC

**Figure 1:**
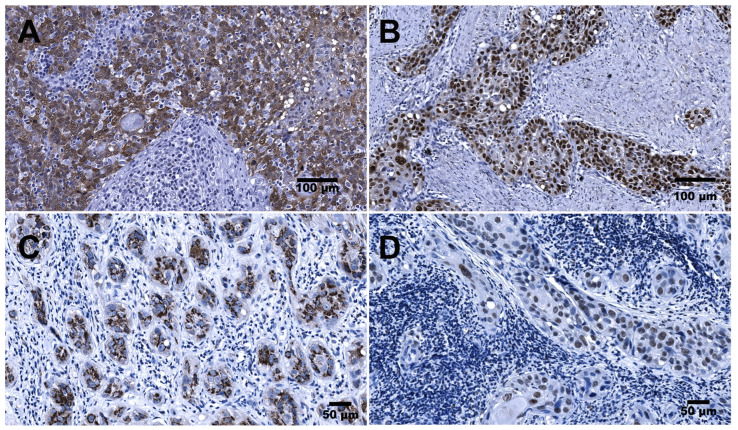
Immunohistochemical detection of p16, p53, HPV E6, and HPV E7 antigens in HNSCC: (**A**) OPSCC (palatine tonsil). Representative image from a case demonstrating >75% p16-positive tumor cells displaying mostly nuclear and cytoplasmic expression. (**B**) LSCC. Representative image of p53 overexpression demonstrating uniform strong nuclear staining of tumor cells. (**C**) OPSCC (palatine tonsil). Representative image demonstrating cytoplasmic expression of HPV16 E6 protein confirmed in tumor cells organized in cords. (**D**) OPSCC (palatine tonsil). Representative image demonstrating nuclear expression of HPV16 E7 protein confirmed in the tumor cells organized as nests and cords. Scale bars: 100 µm and 50 µm.

p16 overexpression (p16+; Figure 1A) was found in 24/106 (22.64%) of the HNSCC samples. More specifically, it was found in 16/34 (47.06%), 6/41 (14.63%), and 2/31 (6.45%) of the OPSCC, LSCC, and HPSCC samples, respectively. Simultaneously, when stratified by HPV16 positivity (HPV16+), p16 overexpression was confirmed in 15/26 (57.69%), 5/22 (22.73%), and 2/20 (10%) of the OPSCC, LSCC, and HPSCC samples, respectively.

### 3.4. IHC Expression of p53 in HNSCC

p53 overexpression (p53+; Figure 1B) was confirmed in 49/106 (46.23%) of the HNSCC samples. More specifically, it was confirmed in 17/34 (50%), 21/41 (51.22%), and 11/31 (35.48%) of the OPSCC, LSCC, and HPSCC samples, respectively. An analysis of the HPV16+ samples showed p53 downregulation (p53−) in 15/26 (57.69%), 10/22 (45.45%), and 14/20 (70%) of the OPSCC, LSCC, and HPSCC samples, respectively. Furthermore, in the E6/E7 mRNA+ samples, p53 downregulation was found in 11/15 (73.33%) of the OPSCC samples and 1/2 (50%) of the LSCC samples.

### 3.5. IHC Expression of HPV16 E6 and E7 Proteins in HNSCC

Overexpression of HPV16 E6 protein (Figure 1C) was immunohistochemically confirmed in 44/106 (41.5%) of the HNSCC samples. More specifically, it was confirmed in 21/34 (61.8%), 14/41 (34.1%), and 9/31 (29.0%) of the OPSCC, LSCC, and HPSCC samples, respectively.

In turn, overexpression of HPV16 E7 protein (Figure 1D) was found in 39/106 (36.8%) of the HNSCC samples. More specifically, it was found in 19/34 (55.9%), 14/41 (24.1%), and 6/31 (19.4%) of the OPSCC, LSCC, and HPSCC samples, respectively.

### 3.6. Kaplan–Meier Survival Analysis

#### 3.6.1. OS and DSS, Depending on HPV DNA (HR-HPV and LR-HPV)

The five-year OS and DSS were assessed in patients who were HPV-positive compared to HPV-negative, depending on the location of the primary tumor.

**Figure 2:**
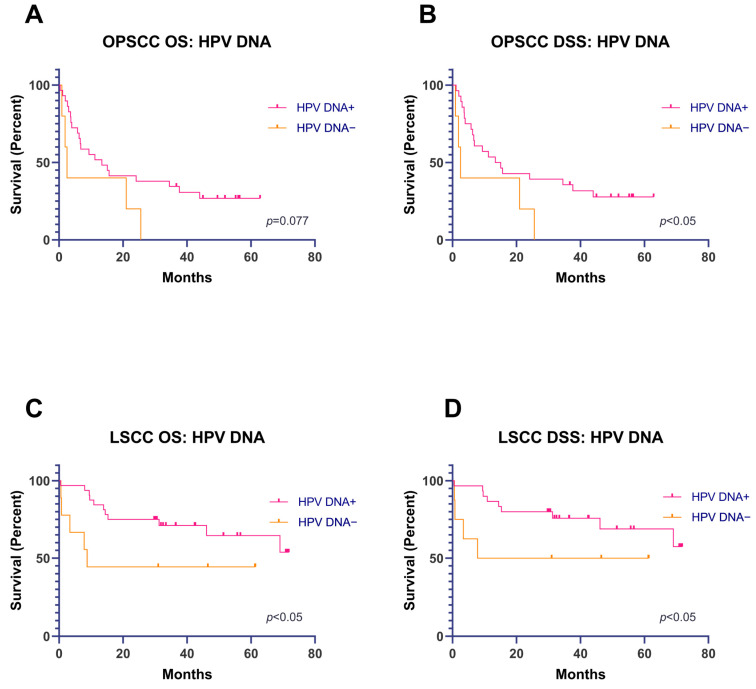
(**A**,**B**) OS and DSS analyses (Kaplan–Meier), depending on the presence of HPV DNA (HR and LR) in OPSCC. (**C**,**D**) OS and DSS analyses (Kaplan–Meier), depending on the presence of HPV DNA (HR and LR) in LSCC.

For the oropharynx, the OS rates were 26.82% and 0% for patients who were HPV-positive and HPV-negative, respectively, although this difference failed to reach statistical significance (*p* = 0.077; Figure 2A). The DSS rates were 27.78% and 0% for these groups of patients (*p* < 0.05; Figure 2B), respectively.

For patients with LSCC, the OS rates were 64.59% and 44.44% in patients who were HPV-positive and HPV-negative (*p* < 0.05; Figure 2C), respectively. The DSS rates were 68.90% and 50% for patients who were HPV-positive and HPV-negative (*p* < 0.05; Figure 2D), respectively.

As all HPSCC samples were HPV DNA+, a Kaplan–Meier survival analysis could not be performed.

#### 3.6.2. OS and DSS, Depending on Immunohistochemical Expression of HPV16 DNA, HPV16 E6/E7 mRNA, and p16, p53, E6, and E7 Proteins

We performed a Kaplan–Meier survival analysis with a stratification of patients depending on the location of the primary tumor. The OS and DSS were calculated. For most variables, a univariate survival analysis with the Kaplan–Meier method failed to reach statistical significance. 

**Figure 3:**
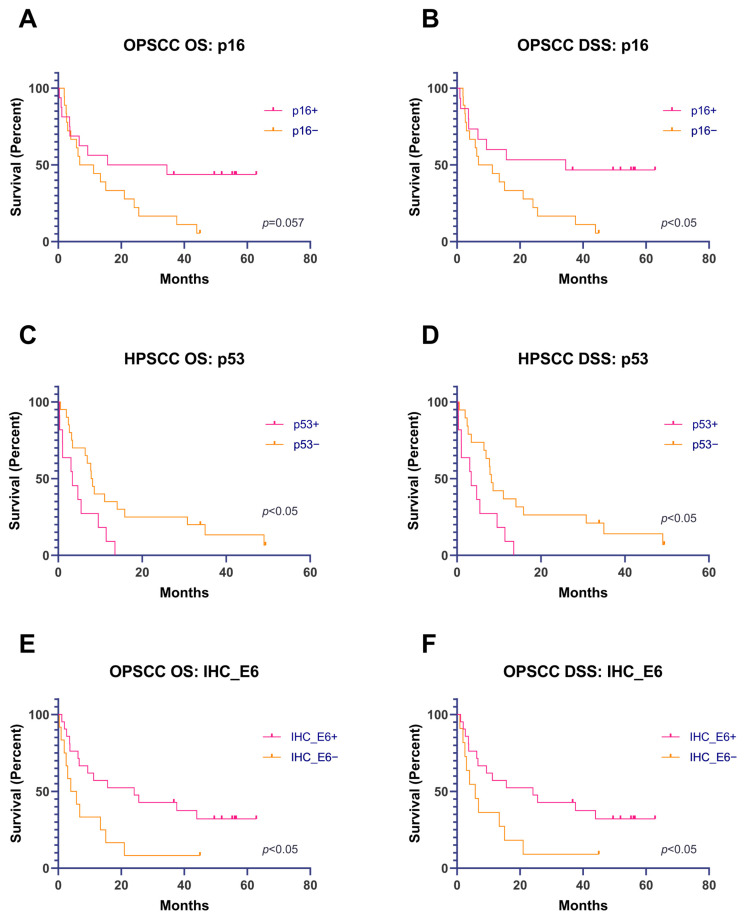
(**A**,**B**) OS and DSS analyses (Kaplan–Meier), depending on the result of the immunohistochemical expression of p16 in OPSCC. (**C**,**D**) OS and DSS analyses (Kaplan–Meier), depending on the results of the immunohistochemical expression of p53 in HPSCC. (**E**,**F**) OS and DSS (Kaplan–Meier), depending on the results of the immunohistochemical expression of HPV16 E6 protein in OPSCC.

There were borderline statistically significant differences (*p* = 0.057, Figure 3A,B) between p16+ and p16− OPSCC for OS and statistically significant differences for DSS.

A Kaplan–Meier survival analysis of p53+ and p53− HPSCC showed statistically significant differences in OS and DSS (Figure 3C,D).

The immunohistochemical overexpression of HPV16 E6 protein was associated with significantly better OS and DSS in patients with OPSCC (Figure 3E,F).

### 3.7. Multivariate Cox Regression Analysis

The age; sex; hazards; applied treatment; immunohistochemical expression of p16, p53, E6 protein, and E7 protein; and the presence of HPV16 DNA and E6/E7 mRNA were included in the Cox model. First, a multivariate survival analysis was performed for all patients with head and neck tumors. Second, each anatomical location of the head and neck cancers was analyzed separately: oropharynx, larynx, and hypopharynx.

#### 3.7.1. All HNSCC

The results of the analysis of all patients with head and neck cancer are summarized in Table 2.

The Cox regression analysis suggested that the T1 stage was associated with a lower risk of early death. While the results for each T stage did not reach statistical significance, there was a trend towards a higher early death risk with a higher T stage; patients with HNSCC with a T4 tumor had a 2.68-fold higher probability of death. The analysis also showed that a higher N stage was associated with a higher risk of early death. The N1 stage (in reference to N0) was associated with a 4.98-fold greater risk of early death, and the risk notably increased in the N3 stage. A lower tumor differentiation grade (G) was associated with a higher risk of early death. Patients with G3 tumors (well-differentiated) had an 81% lower risk of early death than patients with G1 tumors (undifferentiated).

The effect on survival was also statistically significant for treatment. Patients who received a combined treatment (RT+ChT+/−OP) showed a lower risk of early death.

Immunohistochemical overexpression of HPV16 E6 protein (>10%) was associated with a lower hazard ratio (Exp(B) = 0.3492, *p* = 0.0147). Other variables did not show statistical significance.

#### 3.7.2. OPSCC

This group encompassed 34 patients with 26 events (death). Two patients were excluded from the analysis due to missing values. Table 3 depicts the Cox regression results of the variables for OPSCC.

The Cox regression analysis showed that the immunohistochemical expression of p16, p53, and HPV16 proteins E6 and E7; the T grade; the applied treatment; and smoking significantly affected patients’ survival.

**Figure 4:**
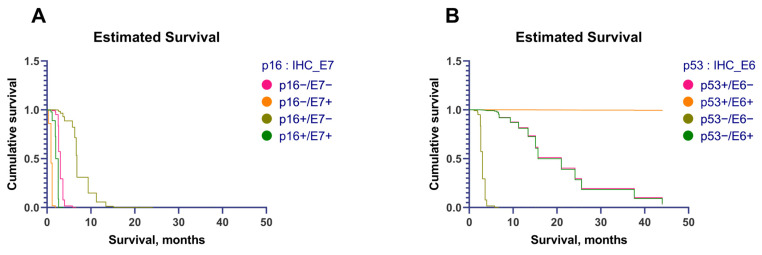
(**A**) Estimated survival, depending on the immunohistochemical expression of p16 and E7 protein. (**B**) Estimated survival, depending on the immunohistochemical expression of p53 and E6 protein.

The overexpression of p16, p53, and HPV16 E6 protein showed much lower hazard ratios and was associated with significantly improved survival. On the contrary, the overexpression of HPV16 E7 protein was associated with a high risk of early death. A graphical analysis showed that the overexpression of p16 (p16+) in a tumor was associated with better survival than that of patients with p16-negative tumors (Figure 4A). However, the overexpression of HPV16 E7 protein was associated with decreased survival. Moreover, when combining the two markers (p16 and HPV16 E7 protein), E7 protein overexpression (E7+) decreased survival, even in patients with p16+ tumors (Figure 4A). Figure 4B shows that the best survival was seen in patients with p53-positive (p53+)/HPV16 E6 protein positive (E6+) tumors and that the worst was seen in patients with p53−/E6− tumors. There was no difference in survival between patients with p53−/E6+ and p53+/E6− tumors.

A larger tumor size (T grade) negatively affected survival. An analysis showed a lower risk of early death for tumors with lower N grades; however, the difference was not statistically significant.

The patients who underwent radiotherapy had a significantly lower risk of early death than patients with other treatment modalities.

#### 3.7.3. LSCC

This group encompassed 41 patients with 16 events (death). A Cox regression model with all included variables was statistically significant (*p* < 0.001). An analysis showed that no variable significantly affected survival. 

#### 3.7.4. HPSCC

This group encompassed 31 patients with 29 events (death). Table 4 depicts the Cox regression analysis for patients with HPSCC.

The Cox regression model showed that the expression of p16 and HPV16 E6 protein; the presence of HPV16 DNA; the hazards; and the T, N, and M grades statistically significantly affected survival. The effects of the other variables were not statistically significant.

**Figure 5:**
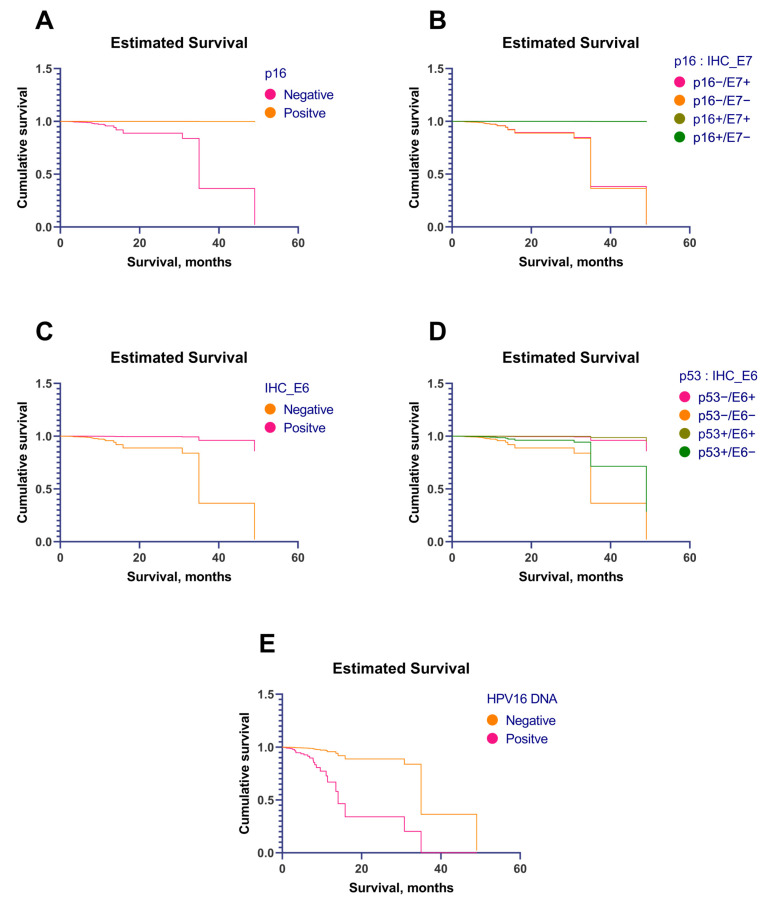
(**A**) Estimated survival (Cox regression), depending on the immunohistochemical expression of p16. (**B**) Estimated survival (Cox regression) depending on the immunohistochemical expression of p16 and HPV16 E7 protein. (**C**) Estimated survival (Cox regression), depending on the immunohistochemical expression of HPV16 E6 protein. (**D**) Estimated survival (Cox regression), depending on the immunohistochemical expression of p53 and HPV16 E6 protein. (**E**) Estimated survival (Cox regression), depending on the presence of HPV16 DNA.

The overexpression of p16 and HPV16 E6 protein was associated with an extremely low risk of early death (Figure 5A,C). By combining the p16 status and the HPV16 E7 protein status, we found that E7 protein expression did not affect survival (Figure 5B, overlaying of the curves). However, combining the p53 and HPV16 E6 protein statuses showed that patients with E6+ tumors had better survival and that p53 overexpression seems to increase survival even more in these patients (Figure 5D). The worst survival was in the group of patients with p53−/E6− tumors.

The Cox regression analysis revealed that larger primary tumors are associated with a higher risk of early death. Patients with T3 tumors had 87% less risk of early death than patients with T4. Moreover, a lower N grade was associated with lower hazard ratios. Lastly, the presence of distal metastases was associated with a 22-fold increase in the risk of death.

Smoking patients had a 57-fold increase in the risk of early death in comparison to non-smokers/non-drinkers.

## 4. Discussion

The current study aimed to assess the roles of HPV infection and associated markers such as p16, p53, HPV16 E6/E7 oncoproteins, the presence of HPV DNA, and E6/E7 mRNA in survival. 

An initial univariate survival analysis (Kaplan–Meier) shows the potential role of not only HR-HPV but also LR-HPV infection in the survival of patients with OPSCC and LSCC, as 1/3 of the patients have a probability of LR-HPV infection. The study results suggest that patients with HPV-DNA-positive OPSCC and LSCC have a better 5-year OS and DSS. These results agree with other studies where patients with HNSCC and patients with tonsil cancer also had better survival rates if the tumors were positive for HPV DNA [38,39]. This is probably due to better radiosensitivity of HPV+ tumors, which means patients could benefit from the “softer” treatment applied to HPV-positive tumors and increases positive outcomes for the patients [38]. On the other hand, HPV-infected cells could be more visible to the host’s immune system, allowing for easier identification as well as the destruction of virus-related tumor tissues. In that case, a deeper investigation of the HPV activity in patients with HNSCC and the interaction with their immune systems would be required. 

It is well documented that patients with HPV-positive OPSCC have higher 3- and 5-year survival rates than patients who are HPV-negative [40], but the consensus is made for HR-HPV (mostly HPV16 and 18). For LSCC, many studies have shown no significant survival increase for HPV-positive tumors [7,41,42]. However, in recent years, there have been studies with results similar to ours, with better survival in patients with HPV-positive LSCC [9,43]. 

On the other hand, in our study, the stratification of patients with HNSCC by the tumor location and the identification of specific HPV types showed that the presence of HPV16 DNA in hypopharyngeal squamous cell carcinoma cases substantially decreased the survival rates of patients. This indicates that HPV16 may play a significant role in HPSCC development. Additionally, the immunological aspects should be considered. The presence of viral antigens could promote anti-tumor immunity and lead to better survival of the patients [44,45,46].

Head and neck cancers encompass a multitude of subsites for cancer development. Sometimes studies analyzing the effects of HPV on the survival of head and neck cancers can be confusing in that they unify the survival analysis without stratifying the primary tumors by location, especially hypopharyngeal and laryngeal cancers, which are sometimes combined in non-oropharyngeal cancers [14,47]. In our view, this could lead to incorrect conclusions. The oropharynx, larynx, and hypopharynx are three distinct locations with different prognoses based on lymphatic drainage alone. In our study, an analysis of all HNSCC in a Cox regression did not show p16, p53, or other variables to be significant factors affecting the survival of the patients. This indicates that patients should preferably be stratified by the primary location of the tumor to obtain a more comprehensive view of the potential risk factors.

This study reaffirmed the predictive role of p16 overexpression in OPSCC (univariate survival analysis), confirming better survival in patients with p16+ tumors [48,49]. This trend continued in the Cox regression analysis, with statistical significance further confirming its role as a distinct predictive marker for OPSCC. However, for HPSCC and LSCC, this could not be confirmed in the univariate survival analysis. The Cox regression analysis showed better survival and a lower risk of death for patients with p16+ HPSCC, suggesting that there might be a reason to consider it as a predictive marker. Several studies have shown similar findings [50,51]. There is also a question of p16’s association with HPV activity in non-oropharyngeal squamous cell carcinoma, whether it can be used as a surrogate marker for HPV infection, and whether it serves as a suitable prognostic factor of survival. Several studies have shown that p16 often does not correspond to the HPV status in non-oropharyngeal cancers; however, it has a prognostic value for survival [52,53,54].

The lack of significance for many analyzed variables in OPSCC (univariate survival analysis) of our study could be due to the relatively small patient number in this subgroup, which could affect the statistical power of analysis. Additionally, the high number of smokers and alcohol abusers could also affect the significance of the results. This is accounted for in the Cox regression model.

The univariate survival analysis of p53 immunohistochemical expression showed significantly better OS and DSS in p53+ HPSCC. The trend persisted in the Cox regression, although without statistical significance. Similar findings were present for OPSCC in the Cox regression analysis; p53 overexpression (p53+) was associated with a significantly lower risk of death. This could be due to the tumor-suppressing properties of p53. However, there was a considerable number of HPV16-positive samples and even more HPV16 E6/E7 mRNA-positive OPSCC samples. A logical picture would be that in HPV-driven cancer, p53 is suppressed, resulting in a p53-negative result that is confirmed using immunohistochemistry. Published data suggest that HPV-driven tumors show p53 downregulation [55,56,57]. On the contrary, Hasegawa et al. [58] reported that p53 overexpression correlates with a better response to chemotherapy and is thus associated with better survival. Similar results were demonstrated by Sun et al. [59]. In these studies, however, the HPV status was not studied. Initially, in HPV-driven cancers, there could be p53 overexpression due to the degradation of pRb by E7 oncoprotein and increased stabilization of p53 [60]. A meta-analysis of oral tongue squamous cell carcinoma showed that p53 could not be used as a prognostic biomarker for these tumors [61]. Similar conclusions were made by Halec et al. for LSCC [36]. Unfortunately, our study did not include an assessment of TP53 gene mutations, which could have clarified some questions about the previously mentioned points [62,63]. Additionally, there is a possibility that p53 overexpression is unrelated to HPV infection, especially considering the high number of smokers in our study. Additional studies are needed to study the prognostic role of p53 in HNSCC, especially in OPSCC and HPSCC.

To the best of our knowledge, very few studies have been focusing on HPV oncoprotein E6/E7 immunohistochemical expression and its role in survival or prognostic values. As E6 and E7 are considered to be the main driving forces of HPV-mediated carcinogenesis, we found it interesting to study the role of these proteins in survival using immunohistochemistry. In the cases of both OPSCC and HPSCC, the immunohistochemistry results of HPV16 E6 protein expression showed that patients with positive staining in their tumor samples had a better survival rate. However, a high expression of either p16 or p53 was simultaneously found with E6, which could be considered a positive outcome marker for the patient. Moreover, there is a possibility that at a certain stage of viral activity, this oncogene (E6) did not have time to disrupt the cell cycle. For example, E6 initiates proteasome-dependent p53 degradation by recruiting the ubiquitin ligase E6AP. Furthermore, only the combined complex of E6 and E6AP is reactive with p53. This means that the expression of a single HPV16 E6 protein cannot affect p53 degradation (detection could be less informative for a patient’s outcome prognosis) [64]. Unfortunately, E6AP activity was not studied in this research. A prospective study (of the dynamics with several time points) might better reveal HPV oncogenes’ roles in the progression of an HNSCC tumor, as a persistent HPV infection is a major factor for carcinogenesis [65]. With this study, it is difficult to distinguish persistent from non-persistent HPV infections (sampling was performed only a single time). However, in patients with HPSCC, E6 protein was detected using only immunostaining, while E6 mRNA was not detected, and HPV16 DNA was still detectable. This could indirectly indicate the presence of a persistent HPV16 infection, which could be one of the reasons why the presence of HPV16 DNA in the samples of patients with HPSCC showed worse outcomes.

E7 is recognized as the major transforming protein of high-risk HPVs due to mutational analyses in transformation assays [66]. In addition, it was shown that E7 precisely drives early tumorigenesis [67]. The present study shows that the IHC overexpression of HPV16 E7 protein in OPSCC is associated with a poorer prognosis (Cox regression). However, in HPV-associated tumors, the E7 protein should be the driving factor for p16 overexpression, which is associated with better survival. On the other hand, some studies report that the overexpression of p16 has consistently and repeatedly been shown to be associated with a better response to therapy and a favorable clinical outcome in OPSCC, and not all cases of p16 overexpression could be related to HPV’s oncogenic activity [68,69]. This suggests the presence of additional mechanisms of E7-protein-associated carcinogenesis. Several studies have shown that E7 induced the upregulation of several types of matrix metalloproteinases [70,71]. This process has been linked to the promotion of the invasiveness the tumors [72]. Additionally, the protein function of HR-HPV E7 has been associated with a more stable mitotic function that is needed for viral genome maintenance and replication [73,74]. These processes could lead to an invasive and potentially metastatic phenotype of cancer, and this could explain the poorer prognosis in OPSCC with IHC HPV16 E7 protein overexpression [66]. Oton-Gonzalez et al. [75] showed that patients with OPSCC with detectable HPV16 E7 protein in their serum had poorer relapse-free survival and OS. The authors also showed a correlation between E7 protein in serum and E7 mRNA expression. Thus, they concluded that the source of the E7 protein must have been HPV16-positive cancer, more specifically circulating tumor cells, suggestive of the metastatic process. It is worth noting that not all tumors are HPV-related, and it was shown that virus-induced oncogenesis takes a long time to develop and that some patients with HNSCC can have a concomitant HPV infection [66].

One limitation of our study is the relatively small number of patients for each region (oropharynx, larynx, and hypopharynx), which could result in insufficient statistical power and limit the conclusions drawn for some markers, especially if they did not reach statistical significance. However, it is hard to deny the observed trends of the studied markers and their effects on survival. The other limitation is that almost all HPSCC samples were FFPE due to possible genetic material degradation, especially that of RNA. On the other hand, all samples were viable for analysis based on the intrinsic control of the kit that was used (mRNA detection) or β-globin detection (DNA quality).

## 5. Conclusions

HPV infection plays a significant role in the tumorigenesis of HNSCC, especially OPSCC. It should be noted that not only HR-HPV but also LR-HPV could affect survival prognosis. The immunohistochemical assessment of HPV16 E6 protein expression should be interpreted as a useful prognostic marker for OPSCC and HPSCC.

## Figures and Tables

**Table 1:** Patients’ characteristics.

	Cases (*n* = 106)		
	OPSCC (*n* = 34)	LSCC (*n* = 41)	HPSCC (*n* = 31)
Sex: Male Female	27 7	39 2	29 2
Age (median)	58.5	64.3	65.9
T grade: T1 T2 T3 T4	6 6 6 16	4 8 24 5	0 4 16 11
N grade: 0 1 2 3	1 15 12 6	4 7 22 8	0 0 10 21
M grade: 0 1	34 0	40 1	27 4
G grade *: 1 2 3	5 21 7	5 34 2	6 21 4
Hazards: None Smoking Smoking and alcohol abuse	9 8 17	4 29 8	3 20 8
Treatment ˆ: RT OP RT+OP RT+ChT (Cetuximab)+/−OP Symptomatic	16 0 2 10 6	1 9 29 0 1	21 0 4 0 6

* One patient had missing value in the OPSCC group. ˆ One patient had a missing value in the LSCC group. RT—radiotherapy, OP—surgery treatment, ChT—chemotherapy.

**Table 2:** Cox regression survival analysis for all HNSCC.

Variables		N = 106 ^$^	β	*p* ˆ	Survival	
Name	Groups *				Hazard Ratios (Exp(β))	95% CI
Sex	Female *	11			(1)	
	Male	92	1.024	0.0627	2.785	0.9886 to 8.688
Age			0.01344	0.4200	1.014	0.9810 to 1.048
p16	Negative *	81			(1)	
	Positive	22	0.5351	0.2412	1.708	0.6693 to 4.081
p53	Negative *	55			(1)	
	Positive	48	0.4982	0.1658	1.646	0.8141 to 3.358
IHC_E6	<10% *	60			(1)	
	>10%	43	−1.052	**0.0147**	0.3492	0.1464 to 0.8037
IHC_E7	<10% *	65			(1)	
	>10%	38	0.4807	0.2956	1.617	0.6590 to 4.024
Hazards	None *	15			(1)	
	Smoking	57	0.5992	0.2624	1.821	0.6636 to 5.526
	Smoking and alcohol abuse	31	−0.1794	0.7552	0.8358	0.2768 to 2.700
Location	Oropharynx *	32			(1)	
	Larynx	40	−0.7745	0.3747	0.4609	0.08271 to 2.545
	Hypopharynx	31	−0.5893	0.3045	0.5547	0.1772 to 1.693
T	1 *	10			(1)	
	2	18	−0.2886	0.6946	0.7493	0.1843 to 3.473
	3	44	0.5419	0.4727	1.719	0.4207 to 8.411
	4	31	0.9882	0.1683	2.686	0.7107 to 12.33
N	0 *	41			(1)	
	1	33	1.607	**0.0011**	4.988	1.944 to 13.55
	2	22	1.372	**0.0182**	3.943	1.277 to 12.62
	3	7	2.208	**0.0036**	9.098	2.042 to 40.88
M	0 *	98			(1)	
	1	5	1.104	0.1662	3.015	0.5851 to 13.67
G	1 *	15			(1)	
	2	76	−0.9494	**0.0413**	0.387	0.1556 to 0.9791
	3	12	−1.658	**0.0058**	0.1906	0.05657 to 0.606
HPV16_DNA	Negative *	37			(1)	
	Positive	66	0.4515	0.2491	1.571	0.7248 to 3.395
HPV16_E6E7_RNA	Negative *	87			(1)	
	Positive	16	−0.9763	0.1399	0.3767	0.09878 to 1.335
Treatment	RT *	37			(1)	
	OP	9	−1.042	0.3186	0.3528	0.03495 to 2.325
	RT+OP	35	−0.6763	0.2432	0.5085	0.1548 to 1.506
	RT+ChT (Cetuximab) +/−OP	9	−2.089	**0.0163**	0.1239	0.01986 to 0.6441
	Symptomatic	13	0.8416	0.0635	2.32	0.9130 to 5.507

* group of reference. ^$^ Three were excluded due to missing values. ˆ statistically significant values (*p* < 0.05) are highlighted in bold.

**Table 3:** Cox regression survival analysis for OPSCC.

Variables		N = 34 ^$^	β	*p* ˆ	Survival	
Name	Groups *				Hazard Ratios (Exp(β))	95% CI
Sex	Female *	7			(1)	
	Male	25	−3.121	0.0810	0.04411	0.001033 to 1.742
Age		32	−0.02128	0.8114	0.9789	0.8275 to 1.177
p16	Negative *	18			(1)	
	Positive	14	−3.548	**0.0532**	0.02879	0.0005461 to 0.9340
p53	Negative *	16			(1)	
	Positive	16	−6.206	**0.0028**	0.002018	1.930 × 10^−5^ to 0.08535
IHC_E6	<10% *	12			(1)	
	>10%	20	−6.171	**0.0265**	0.002089	1.830 × 10^−6^ to 0.1431
IHC_E7	<10% *	14			(1)	
	>10%	18	6.154	**0.0355**	470.6	8.716 to 604,132
Hazards	None *	8			(1)	
	Smoking	8	8.18	**0.0323**	3568	3.203 to 10,181,954
	Smoking and alcohol abuse	16	5.424	0.0801	226.9	0.4392 to 139,247
T	1	6	−4.794	**0.0137**	0.008275	0.0001011 to 0.2757
	2	6	−7.933	**0.0010**	0.0003588	1.456 × 10^−6^ to 0.02453
	3	4	−5.286	**0.0480**	0.00506	5.478 × 10^−6^ to 0.2114
	4 *	16				
N	0	1	−29.16	>0.9999	2.166 × 10^−13^	-
	1	13	0.5427	0.7926	1.721	0.02756 to 200.9
	2	12	−3.366	0.1093	0.03453	0.0001571 to 1.714
	3 *	6			(1)	
M	0	32	-	-	-	-
G	1 *	5			(1)	
	2	21	1.356	0.4788	3.882	0.08016 to 198.0
	3	6	−0.8802	0.6145	0.4147	0.007811 to 11.00
HPV16_DNA	Negative *	8			(1)	
	Positive	24	1.07	0.4826	2.914	0.1090 to 47.14
HPV16_E6E7_RNA	Negative *	18			(1)	
	Positive	14	−1.53	0.3384	0.2166	0.003954 to 4.418
Treatment	RT *	15			(1)	
	OP	0	-	-	-	-
	RT+OP	2	8.757	**0.0100**	6352	7.160 to 9,678,504
	RT+ChT (Cetuximab) +/−OP	9	1.005	0.6443	2.731	0.06476 to 538.3
	Symptomatic	6	9.218	**0.0003**	10072	154.8 to 6,028,349

* group of reference. ^$^ Two were excluded due to missing values. ˆ Statistically significant values (*p* < 0.05) are highlighted in bold.

**Table 4:** Cox regression survival analysis for HPSCC.

Variables		N = 31	β	*p* ˆ	Survival	
Name	Groups *				Hazard Ratios (Exp(β))	95% CI
Sex	Female *	2			(1)	
	Male	29	1.92	0.2873	6.823	0.2575 to 478.6
Age		31	0.0571	0.2702	1.059	0.9605 to 1.194
p16	Negative *	29			(1)	
	Positive	2	−6.638	**0.0049**	0.001309	5.631 × 10^−6^ to 0.08768
p53	Negative *	20			(1)	
	Positive	11	−1.099	0.2540	0.333	0.04332 to 2.109
IHC_E6	<10% *	22			(1)	
	>10%	9	−3.211	**0.0108**	0.04033	0.002158 to 0.3739
IHC_E7	<10% *	25			(1)	
	>10%	6	−0.04985	0.9711	0.9514	0.04166 to 10.39
Hazards	None *	3			(1)	
	Smoking	20	4.049	**0.0214**	57.36	2.263 to 3407
	Smoking and alcohol abuse	8	−0.8085	0.6127	0.4455	0.01394 to 9.424
T	1	0	-	-	-	-
	2	4	2.196	0.0950	8.986	0.7027 to 155.3
	3	16	−2.026	**0.0240**	0.1319	0.02240 to 0.7996
	4 *	11			(1)	
N	0 *	6			(1)	
	1	16	−2.825	**0.0421**	0.05932	0.003106 to 0.8054
	2	8	−2.719	**0.0235**	0.06597	0.005426 to 0.6552
	3	1	4.872	**0.0108**	130.6	2.628 to 7490
M	0 *	27			(1)	
	1	4	3.091	**0.0274**	21.99	1.535 to 460.4
G	1 *	6			(1)	
	2	21	−2.035	0.0912	0.1307	0.01189 to 1.553
	3	4	−2.087	0.1338	0.124	0.006553 to 1.883
HPV16_DNA	Negative *	11			(1)	
	Positive	20	2.205	**0.0194**	9.071	1.578 to 70.34
HPV16_E6E7_RNA	Negative *	31	-	-	-	-
	Positive	0	-	-	-	-
Treatment	RT *	21			(1)	
	OP	0	-	-	-	-
	RT+OP	4	1.563	0.1378	4.771	0.5985 to 42.89
	RT+ChT (Cetuximab) +/−OP	0	-	-	-	-
	Symptomatic	6	0.17	0.8610	1.185	0.1367 to 7.244

* group of reference. ˆ Statistically significant values (*p* < 0.05) are highlighted in bold.

## Data Availability

The results related to the immunohistochemical assessment of p16, viral proteins HPV16 E6 and E7, and HPV DNA testing in the samples obtained from patients with hypopharyngeal and laryngeal cancer were published in Viruses (https://doi.org/10.3390/v13061008). The aforementioned article aimed to estimate the prevalence of aforementioned markers in laryngeal and hypopharyngeal cancers.
